# The Role of Disordered Regions in Orchestrating the Properties of Multidomain Proteins: The SARS-CoV-2 Nucleocapsid Protein and Its Interaction with Enoxaparin

**DOI:** 10.3390/biom12091302

**Published:** 2022-09-15

**Authors:** Marco Schiavina, Letizia Pontoriero, Giuseppe Tagliaferro, Roberta Pierattelli, Isabella C. Felli

**Affiliations:** Magnetic Resonance Center (CERM) and Department of Chemistry “Ugo Schiff”, University of Florence, Via L. Sacconi 6, 50019 Sesto Fiorentino, Italy

**Keywords:** SARS-CoV-2, COVID-19, IDP, viral proteins, enoxaparin, NMR

## Abstract

Novel and efficient strategies need to be developed to interfere with the SARS-CoV-2 virus. One of the most promising pharmaceutical targets is the nucleocapsid protein (N), responsible for genomic RNA packaging. N is composed of two folded domains and three intrinsically disordered regions (IDRs). The globular RNA binding domain (NTD) and the tethered IDRs are rich in positively charged residues. The study of the interaction of N with polyanions can thus help to elucidate one of the key driving forces responsible for its function, i.e., electrostatics. Heparin, one of the most negatively charged natural polyanions, has been used to contrast serious cases of COVID-19 infection, and we decided to study its interaction with N at the molecular level. We focused on the NTR construct, which comprises the NTD and two flanking IDRs, and on the NTD construct in isolation. We characterized this interaction using different nuclear magnetic resonance approaches and isothermal titration calorimetry. With these tools, we were able to identify an extended surface of NTD involved in the interaction. Moreover, we assessed the importance of the IDRs in increasing the affinity for heparin, highlighting how different tracts of these flexible regions modulate the interaction.

## 1. Introduction

Since the COVID-19 pandemic impacted our lives, the development of novel and robust pharmacological strategies to contrast the SARS-CoV-2 virus became a priority worldwide. This pushed biomedical researchers to explore different alternatives to face the spreading of the infection [1]. The main results were the development of innovative mRNA-based vaccines and the use of monoclonal antibodies as a therapy [2,3]. These techniques have been fundamental to smoothing out the emergency. Nevertheless, the circulation of the virus is not over yet, and drug discovery studies are continuously in progress.

Nowadays, the most common pharmaceutical approaches target the Spike protein (S) [4], which is the access key to the host’s cells. It is strongly affected by mutations [5,6], some of which are of concern since they affect the transmissibility and antigenicity of the disease. However, other viral proteins have emerged as potential drug candidates and they are now under investigation [7,8]. One of the most promising targets is the Nucleocapsid protein (N), the most expressed protein within the SARS-CoV-2 proteome [9]. 

**Figure 1:**
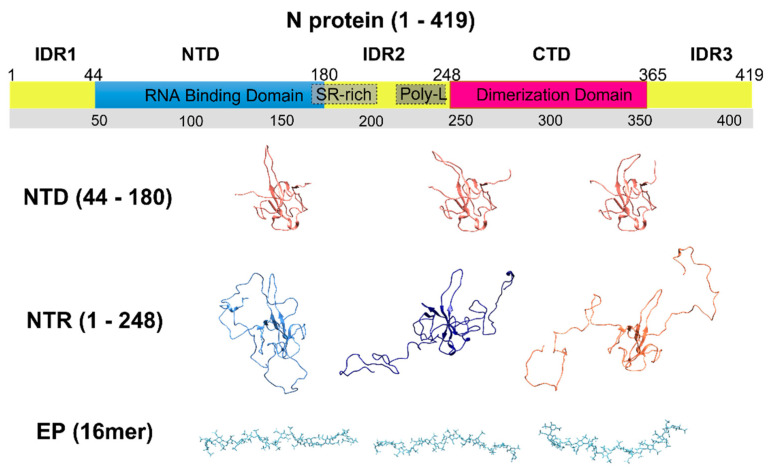
The scheme reported on top shows the modular organization of the nucleocapsid protein [12,13,14,15]. The IDRs are colored in orange (IDR1, IDR2, and IDR3), the NTD is in blue, and the CTD is in red. Some regions of IDR2 important for the discussion are also highlighted (SR-rich; Poly-L). The molecules studied in the present work (NTD, NTR, and EP) are illustrated below the scheme. Three NTD conformers from the 6YI3 [22] PDB entry were selected to show the structural heterogeneity adopted by some parts of NTD. Several NTR conformers were generated using the EOM software (version 3.0. EMBL, Hamburg, Germany) [32,33] based on the NTD conformers. Three of them are reported here to sketch the conformational space that can be sampled by the protein. The EP conformers were selected from the PDB entry 3IRI [34].

N shares 90% of its homology with related proteins from other coronaviruses, and its mutations occur in limited regions of the sequence [8,10]. The main function of N is to package genomic RNA, but it is also involved in pivotal mechanisms for the viral replication cycle [11]. This multi-functional role is possible thanks to the modular organization of its structure (Figure 1). N is composed of two folded domains (N-terminal Domain, NTD, and C-terminal Domain, CTD) and three intrinsically disordered regions (IDR1, IDR2, and IDR3) [12,13,14,15]. These latter portions are necessary both for the formation of the RiboNucleoProtein (RNP) complex and for recruiting partners necessary for the transcription of the viral genome [16,17,18,19]. While the importance of the flexible regions for N protein function has long been recognized [16], their atomic resolution investigation still remains a challenge, in particular, when IDRs are part of a multidomain protein [20]. NMR resonance assignments of the first two IDRs (IDR1 and IDR2) have recently become available [15,21], opening the way to the investigation at atomic resolution of their role in modulating protein function [17,18,19,22,23,24,25]. 

Both the globular RNA binding domain (NTD) and the tethered IDRs are rich in positively charged residues that drive the interaction between the N protein and its partners, like the negatively charged RNA fragments [17,19,22,23,24,25]. The study of the interaction of N with molecules that mimic nucleic acids’ charge, such as polyanions, can thus help to elucidate one of the key driving forces responsible for its function, electrostatic contribution. The aim of this study is, thus, to investigate the interaction of N with one of the naturally occurring polyanions, heparin. This is a ubiquitous linear glycosaminoglycan (GAG), characterized by different degrees of sulfation which confer it a high negative charge. It is a component of the cell surface and of the extracellular matrix. It is also often used as a drug for its anticoagulant properties.

Low molecular weight heparin is used in clinical protocols to contrast serious cases of COVID-19 infection [26]. The literature reports about the interaction between heparin and the N protein detected in human samples, such as blood and saliva [27], and heparin-based resins have been used for hemofiltration in crucially ill COVID-19 patients, demonstrating a reduction in the N blood concentration after the treatment [28]. Furthermore, it was recently shown that N is not strictly confined to the cytosol but it is also found on the infected/transfected cells’ surface, where it binds the heparin of the extracellular matrix [29]. Other viral RNA-binding proteins were found to adopt similar mechanisms to the N one [30,31]; it is suggested that N exploits these properties to interfere with the binding of cytokines to the GAGs.

On these grounds, we studied the interplay between two different N protein constructs and enoxaparin (EP, 16mer, 4.5 kDa, Figure 1), a low molecular weight heparin. In particular, we focused on the N-terminal region of the protein using a construct that comprises residues 1-248 (IDR1-NTD-IDR2, referred to as NTR), and on the NTD construct, (residues 44-180). The two different protein constructs thus differ in the presence of the two positively charged disordered regions that are expected to be relevant for the protein behavior in binding highly negatively charged partners and are central in the present study. We characterized the NTD and NTR interaction with EP using different Nuclear Magnetic Resonance (NMR) approaches and we complemented the analysis with Isothermal Titration Calorimetry (ITC). The high-resolution mapping of the binding obtained in this work could help the design of tailored polyelectrolytes able to interfere with N protein function.

## 2. Materials and Methods

### 2.1. Protein Sample Preparation

The NTD and NTR samples were prepared as previously described [35] and briefly summarized hereafter.

The sequence of the NTD (44-180) was based on SARS-CoV-2 NCBI reference genome entry NC_045512.2, identical to GenBank entry MN90894 [36]. The gene inserted into pET28a(+) containing an N-terminal His6-tag, a tobacco etch virus (TEV) cleavage site vector, was kindly provided by Prof. Fabio Almeida from the University of Rio De Janeiro. After proteolytic TEV cleavage, the produced 14.85 kDa protein does not contain any artificial residue.

Uniformly ^15^N-labeled and ^13^C, ^15^N-labeled NTD was expressed in *E. coli* strain BL21 (DE3) in M9 minimal medium containing 1.0 g/L ammonium chloride (^15^NH_4_Cl) (Cambridge Isotope Laboratories, Tewksbury, MA, USA) and, for ^13^C labeling, 3 g/L ^13^C_6_-D-glucose (Eurisotop, Cambridge Isotope Laboratories, Tewksbury, MA, USA). Protein expression was induced at an Optical Density measured at 600 nm (OD_600_) of 0.7 with 0.2 mM isopropyl-beta-thiogalactopyranoside (IPTG) for 18 h at 16 °C. Cell pellets were resuspended in 50 mM 2-amino-2-(hydroxymethyl)-1,3-propanediol (TRIS) at pH 8.0, 500 mM sodium chloride (NaCl), 20 mM imidazole, 10% *v*/*v* glycerol, and a protease inhibitor cocktail (SIGMAFAST). The cells were disrupted by sonication. The supernatant was cleared by centrifugation for 30 min at 30,000× *g* at 4 °C.

The cleared supernatant was passed over a Ni^2+^-NTA HisTrap HP (GE Healthcare, Chicago, IL, USA), and the His_6_-Trx-tag was cleaved overnight at 4 °C with 1:10 *v*/*v* of TEV protease:protein solution, while dialyzing into fresh buffer composed of 50 mM TRIS at pH 8.0, 500 mM NaCl, and 1 mM dithiothreitol (DTT). TEV protease and the cleaved tag were removed via a second Ni^2+^-NTA HisTrap HP. The fractions containing the pure NTD protein were determined by SDS-PAGE, pooled, and concentrated. Buffer exchange was performed through a PD-10 desalting column (GE Healthcare) or through dialysis, with a final buffer containing 25 mM potassium phosphate (KH_2_PO_4_/K_2_HPO_4_) 150 mM potassium chloride (KCl), and 0.02% sodium azide (NaN_3_) at pH 6.5.

For the NTR (1-248), the gene of the N protein construct comprising residues 1-248 was designed based on the boundaries determined from the SARS-CoV homologue. The codon-optimized gene was synthesized by Twist Bioscience and cloned into the pET29b(+) vector between NdeI and XhoI restriction sites.

Uniformly ^15^N and ^13^C, ^15^N-labelled NTR protein was expressed in *E. coli* strain BL21 (DE3) following the Marley protocol [37]. The cells were grown in 1 L of Luria Bertani medium at 37 °C until OD_600_ of 0.8. Then, the culture was transferred in 250 mL of labeled M9 minimal medium supplemented with 1.0 g/L ^15^NH_4_Cl and, for ^13^C labeling, 3.0 g/L of ^13^C_6_-D-glucose. After 1 h of unlabeled metabolite clearance, the culture was induced with 0.2 mM IPTG at 16 °C for 18 h. The pellet was harvested and stored at −20 °C overnight. The cell pellet was then dissolved in 25 mM TRIS, 1.0 M NaCl, 10% *v*/*v* glycerol, and protease inhibitor cocktail (SIGMAFAST) at pH 8.0 and centrifuged at 30,000× *g* for 50 min at 4 °C.

The soluble fraction was dialyzed overnight against a solution of 25 mM TRIS, pH 7.2, at 4 °C. The protein solution was then loaded onto a HiTrap SP FF 5 mL column and eluted with a 70% gradient of 25 mM TRIS and 1.0 M NaCl. Fractions containing the protein were pooled, concentrated, and loaded onto a HiLoad 16/1000 Superdex 75 pg column equilibrated with 25 mM (KH_2_PO_4_/K_2_HPO_4_), 150 mM KCl, and 0.02% NaN_3_ at pH 6.5. 

Regarding the NTD construct, ^1^H detected experiments were acquired using a 500-µL-sample of 70 µM ^15^N-labelled NTD protein. The titration was performed in 5 mm NMR tubes. Proper aliquots of a 22 mM stock solution of commercially available enoxaparin sodium salt (CLEXANE, Sanofi S.p.A.) were added to the protein solution to reach NTD:EP ratios of 1:0.01, 1:0.025, 1:0.10, 1:0.30, 1:0.60 1:0.90, 1:1.20, 1:2.40, 1:4.80, 1:9.60, and 1:19.20.

Briefly, ^13^C detected experiments were acquired using a 500-µL-sample of 200 µM ^13^C-^15^N-labelled NTD protein. The titration was performed in 5 mm NMR tubes. Proper aliquots of the stock solution of EP were added to the protein solution to reach NTD:EP ratios of 1:0.10, 1:0.30, 1:0.45, 1:0.60 1:0.9, 1:1.20, 1:2.4, and 1:4.8. Moreover, 2D HN experiments were also collected during this titration as control.

Furthermore, ^15^N Relaxation experiments were acquired using a 500-µL-sample of 200 µM ^15^N-labelled NTD protein. The same experiments were recorded after the addition of 1.2 EP equivalents.

Regarding the NTR construct, ^1^H and ^13^C detected experiments were acquired using a 500-µL-sample of 70 µM ^13^C, ^15^N-labelled NTR protein. The titration was performed in 5 mm NMR tubes. Proper aliquots of the stock solution of EP were added to a protein solution sample to reach NTR:EP ratios of 1:0.10, 1:0.30, and 1:1.00.

Moreover, ^1^H detected experiments were repeated using a 500-µL-sample of 70 µM ^15^N-labelled NTR protein. The titration was performed in 5 mm NMR tubes. Proper aliquots of the stock solution of EP were added to a protein solution sample to reach NTR:EP ratios of 1:0.01, 1:0.05, 1:0.10, 1:0.30, 1:0.60, 1:1.20, and 1:6.00.

Diffusion Orderd SpectroscopY (DOSY) experiments were acquired using a 500-µL-sample of 70 µM ^15^N-labelled NTD protein. The same experiments were recorded after the addition of 9.6 EP equivalents.

### 2.2. NMR Experiments

The interaction between the N constructs and EP was followed at 298 K, exploiting a series of 2D HN HSQC [38], 2D HC HSQC [38,39], 2D CACO [40], 2D (H)CBCACO [40], 2D (HCA)CON [41], and mr_HN//CON [42] experiments.

The following spectrometers (Bruker, Billerica, MA, USA) were used:

- a Bruker AVANCE III spectrometer operating at 950.20 MHz ^1^H, 238.93 MHz ^13^C, and 96.28 MHz ^15^N frequencies, equipped with a cryogenically cooled probe head optimized for ^1^H-direct detection (TCI). Namely, *950*.
- a Bruker AVANCE NEO spectrometer operating at 700.06 MHz ^1^H, 176.03 MHz ^13^C, and 70.94 MHz ^15^N frequencies equipped with a cryogenically cooled probe head optimized for ^13^C-direct detection (TXO). Namely, *700C*.
- a Bruker Avance NEO spectrometer operating at 700.13 MHz ^1^H, 176.05 MHz ^13^C, and 70.94 MHz ^15^N equipped with a cryogenically cooled triple resonance probe head optimized for ^1^H-direct detection (TXI). Namely, *700H*.
- a Bruker AVANCE III-HD spectrometer operating at 600.13 MHz ^1^H, 120.90 MHz ^13^C, and 60.81 MHz ^15^N frequencies equipped with a probe head optimized for ^1^H-direct detection (TXI). Namely, *600.*

Standard radiofrequency pulses were used. The decoupling of ^1^H and ^15^N was achieved with waltz65 and garp4 decoupling sequences, respectively [43,44]. All gradients employed had a smoothed square shape.

The 2D HN HSQC [38] experiments recorded to follow the titration of NTD and NTR with EP were acquired at *950*. The carrier frequency for ^1^H was set at 4.7 ppm; for ^15^N, the carrier was set at 120 ppm for standard HN spectra and at 80 ppm for spectra tailored to detect the arginine side-chain’s correlations.

The 2D HC HSQC [38,39], 2D CACO [40], 2D (H)CBCACO [40], and 2D (HCA)CON [41] experiments were acquired at *700C*. Briefly, ^13^C pulses were centered at 176.7 ppm, 49.7 ppm, 45.7 ppm, and 122.7 ppm for the C’, C^α^, C^ali^, and C^aro^ regions. Further, ^15^N pulses were given at 121.0 ppm. The ^1^H carrier was placed at 4.7 ppm. Q5- and Q3-shaped pulses [44] of durations of 300 and 231 μs, respectively, were used for ^13^C band-selective π/2 and π flip angle pulses, except for the π band-selective pulses on the C^α^ region (Q3, 1200 μs) and for the adiabatic π pulse to invert both C’ and C^α^ (smoothed chirp 500 μs, 20% smoothing, 80 kHz sweep width, 11.3 kHz radio frequency field strength).

The interaction between ^13^C- and ^15^N-labelled NTR and EP were followed, exploiting a series of mr_CON//HN [42] experiments acquired at *700C.* The ^13^C pulses were centered at 176.7 ppm and 55.9 ppm for C’ and C^α^. Further, ^15^N pulses were centered at 122.5 ppm for the CON experiment and at 118 ppm for the HN one. The ^1^H carrier, shapes, and duration of the ^13^C selective pulses were the same as reported for the experiments acquired on NTD.

The mr_CON//HN [42] was acquired with an interscan delay of 1.9 s; the HN was acquired within this delay. For each increment of the CON experiment, the in-phase (IP) and antiphase (AP) components were acquired and properly combined to achieve IPAP [45] virtual decoupling. In the mr_CON//HN [42] experiment, solvent suppression was achieved through the 3:9:19 pulse scheme [46].

The acquisition parameters are reported in Table 1.

To complete the available NTD assignment (BMRB 34511 [22]), a 3D (H)CBCACON experiment [41] was also performed on a 450 μM ^13^C,^15^N NTD sample at *950*. Pulses were centered at 176.2 ppm, 56.1 ppm, 45.7 ppm, 122.0 ppm, and 4.7 for C’, C^α^, C^ali^, N, and H regions, respectively. Q5- and Q3-shaped pulses [44] of durations of 259 and 162 μs, respectively, were used for ^13^C band-selective π/2 and π flip angle pulses, except the adiabatic π pulse to invert both C’ and C^α^ (smoothed chirp 500 μs, 20% smoothing, 80 kHz sweep width, 11.3 kHz radio frequency field strength). 

The 3D (H)CBCACON was acquired with an interscan delay of 1.1 s. This spectrum was acquired with 16 scans, with sweep widths of 9566 Hz (^13^C’) × 4830 Hz (^15^N) × 19,118 Hz (^13^C^ali^) and 1024 × 64 × 96 real points in the three dimensions, respectively. The obtained resonances’ assignment is reported in Supplementary Table S1 and deposited in BMRB (51,620) together with the rest of the assignment obtained in our experimental condition.

The NMR experiments to determine the ^15^N relaxation values [38,47] (^15^N *R*_1_, ^15^N *R*_2_, and ^1^H-^15^N NOEs) were recorded at *700H*. The ^15^N R_1_ and R_2_ experiments were performed using the standard Bruker pulse sequences, with 16 scans and sweep widths of 10,869 Hz (^1^H) × 2551 Hz (^15^N) acquiring 2048 × 192 real points in the two dimensions. A relaxation delay of 3.0 s has been used. To determine the ^15^N R_1_ values, the following delays were used: 20 ms, 100 ms, 200 ms, 300 ms, 400 ms, 500 ms, 600 ms, 800 ms, 1000 ms, 1200 ms, 1500 ms, and 2000 ms. The 200 ms point was acquired twice for statistical analysis. To determine the ^15^N R_2_ values, the following delays were used: 16 ms, 32 ms, 48 ms, 64 ms, 80 ms, 96 ms, 112 ms, 128 ms, 160 ms, 192 ms, 240 ms, and 320 ms. The 32 ms point was acquired twice for statistical analysis. The ^1^H–^15^N NOE experiments were performed with 96 scans with sweep widths of 10,869 Hz (^1^H) × 2551 Hz (^15^N) and 2048 × 128 real points in the two dimensions. A relaxation delay of 6.0 s was used.

DOSY experiments were performed at *600*. The stimulated echo version [48] has been exploited using bipolar gradient pulses for diffusion. Solvent suppression was achieved through the 3:9:19 pulse scheme [46]. Both the experiments conducted in the presence and absence of 1.2 equivalents of EP were acquired with an interscan delay of 3.8 s. The gradient distance ∆ was set to 150 ms, and the bipolar gradient length δ was set to 3 ms. The gradient ramp was linear with 128 steps applying a gradient strength from 2% to 95%, with a full power strength of 5.65 G/mm.

All the spectra were processed with TopSpin 4.0.6 and analyzed using CARA [49] and its tool, NEASY [50].

Chemical shifts were referenced using the ^1^H and ^13^C shifts of DSS. The ^15^N chemical shifts were referenced indirectly [51].

### 2.3. Kd Estimation

The dissociation constant (*K_d_*) for the interaction between the two N constructs and EP was determined through NMR spectroscopy measuring the variation of chemical shift for each peak in a series of ^1^H-^15^N HSQC spectra recorded at increasing concentrations of EP. The data were fitted using the following equation:

$$\frac{\Delta_{obs}}{\Delta_{max}}=\frac{C_{P}+C_{EP}+K_{d}-\sqrt{{\left(C_{P}+C_{EP}+K_{d}\right)}^{2}-4C_{P}\cdot C_{EP}}}{2C_{P}}$$

where $\Delta_{obs}$ is the observed chemical shift perturbation at the different titration points, $\Delta_{max}$ is the maximum value obtained at the end of the titration, $C_{P}$ is the total protein concentration (NTD or NTR), $C_{EP}$ is the EP concentration at the different titration points, and $K_{d}$ is the dissociation constant. 

The CSP values of those peaks displaying a perturbation higher than the average were used as inputs in the calculation to estimate the *K_d_*. The residues used to calculate the *K_d_* for the NTD construct were A50, T57, R92, G96, G97, K102, W108, T166, Y172, and A173. The residues used to calculate the *K_d_* for the NTR construct were K38, L45, S176, S180, S183, S194, and T205.

A *K_d_* for the NTR:EP was obtained from isothermal calorimetry (ITC) as well. An NTR sample of 30 µM was dialyzed overnight against the working buffer (25 mM KH_2_PO_4_/K_2_HPO_4_, 150 mM KCl, pH 6.5). The same buffer was used to prepare a batch of EP 300 µM that was used to titrate the protein. Measurements were carried out with a VP-ITC microcalorimeter instrument (MicroCal, Inc., GE Healthcare, Chicago, IL, USA) at 298 K and analyzed using the ITC version of Origin 7.0 with embedded calorimetric fitting routines.

### 2.4. Protein-Ligand Docking

We performed the molecular docking of EP and NTD using the HADDOCK server (version 2.4 Bonvin Lab, Utrecht, The Netherlands) [52,53]. The protein structural coordinates used as input were obtained by selecting one of the models deposited in the Protein Data Bank (PDB) under access code 6YI3 [22]. Protonation states of histidine residues 59 and 145 at pH 7.0 were set accordingly to the HADDOCK standard protocol.

The EP structural coordinates have been derived from the PDB under the access code 3IRI [34]. We selected one of the models, properly renumbering and renaming the different atoms to encode 10 monomers according to HADDOCK’s formalism. 

The protein active residues were selected as those showing a CSP upon interaction with EP, taking into consideration all the acquired spectra (49, 50, 56, 57, 58, 59, 60, 62, 63, 88, 92, 93, 94, 96, 97, 98, 99, 100, 101, 102, 103, 104, 107, 108, 154, 162, 165, 166, 167, 169, 172, 173, and 174). The passive residues were automatically selected by the HADDOCK server.

In addition, NTD’s flexible region composing the “finger” (92–106) was defined as a fully flexible segment for the advanced stages of the docking calculation.

In total, 1000 complex structures of rigid-body docking were calculated by using the standard HADDOCK protocol with an optimized potential for liquid simulation parameters (OPLSX). The final 200 lowest-energy structures were selected for subsequent explicit solvent (water) and semi-flexible simulated annealing.

The final structures were clustered using the fraction of common contacts (FCC) with a cutoff of 0.6 and a minimal cluster size of five.

The 191 resulting structures were sorted into six clusters and the one with the best HADDOCK score was selected for the analysis as discussed later. The latter is composed by 130 structures (68%) while the other clusters contain, respectively 26 (13%), 9 (5%), 14 (7%), 7 (4%), and 5 (3%) structures.

## 3. Results

Different NMR approaches were used to focus on the globular domain (NTD) and on the disordered regions (IDRs) present in the NTR construct. These allowed us to achieve atom-resolved information on the interaction with EP, as described in detail hereafter.

### 3.1. The Interaction of EP with NTD

**Figure 2:**
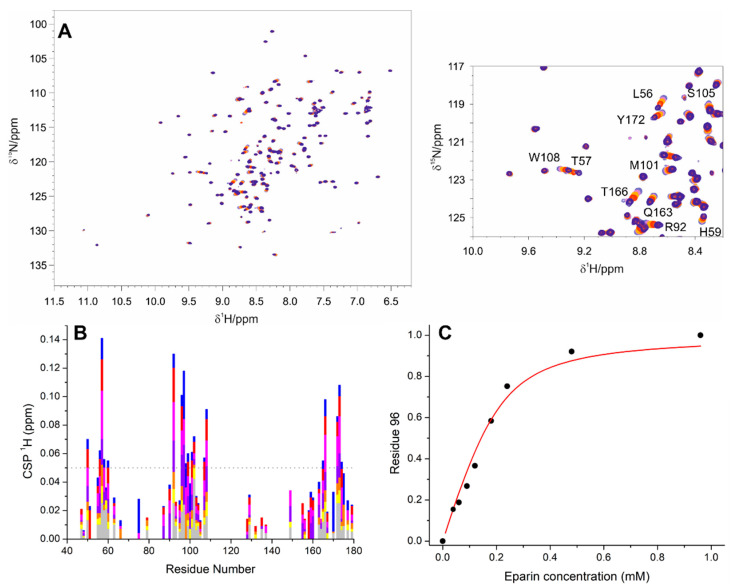
Panel (**A**) reports the overlay of NTD 2D HN spectra upon the addition of EP. Light blue, pink, orange, red, and blue represent the 1:0, 1:0.3, 1:0.6, 1:1.2, and 1:2.40 molar ratios of NTD:EP, respectively (the protein concentration was 200 µM). A zoom in a spectral region where several peaks are perturbed is reported on the right. The assignment of the most perturbed peaks is shown. Panel (**B**) reports the variations in chemical shifts of ^1^H nuclei (CSP) against the residue number at 1:0.30, 1:0.45, 1:0.60, 1:0.90, 1:1.20, 1:2.40, and 1:4.80 of the NTD:EP ratios (grey, yellow, orange, violet, magenta red, and blue, respectively). Panel (**C**) reports the fittings and the obtained *K_d_* values for G96.

Two-dimensional NMR spectra were used to identify at atomic resolution, which are the regions of the protein that are perturbed upon the addition of increasing amounts of EP. As a first step to characterize this interaction, we decided to focus on the NTD construct following changes in the 2D ^1^H-^15^N HSQC NMR spectra (2D HN hereafter) upon the addition of EP. The results are reported in Figure 2A. The interaction is in a fast exchange regime on the NMR time scale, and the observed spectral changes upon the addition of up to 4.8 equivalents of EP to the protein are plotted in Figure 2B. Monitoring the ^1^H chemical shift values upon titration (Figure 2B) allowed us to estimate a dissociation constant (*K_d_*) of 44 ± 9 µM (G96; Figure 2C). 

**Figure 3:**
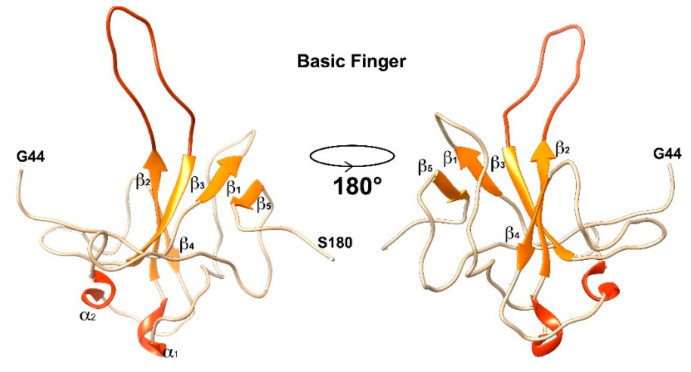
Representation of the secondary structural elements forming the fold of the NTD. β sheets are reported in orange together with the loop composing the finger. α helices are reported in red. The elements that are not comprised in any secondary structural element are reported in grey.

As extensively discussed in the literature [22,24] and shown in Figure 3, NTD is organized into five β-strands (β1, β2, β3, β4, and β5), two short α-helices (α1 and α2), and a flexible hairpin. The secondary structural elements β2–β3 compose the core of the protein fold, very rich in aromatic residues, and extend into the flexible hairpin (the “finger”), rich in positively charged residues. The antiparallel β-sheet formed by β1–β5 is, instead, a junction between the two domain’s ends.

Looking at the ^1^H^N^ chemical shift, the most perturbed residues upon EP interaction are clustered mainly in two regions: the basic finger (R92, G96, G97, D98, G99, M101, and K102) and the β1–β5 antiparallel sheet (L56, T57, Q58, G60, Y172, A173, and E174). Other few residues external to these regions (A50, R107, W108, T165, and T166) were also affected.

**Figure 4:**
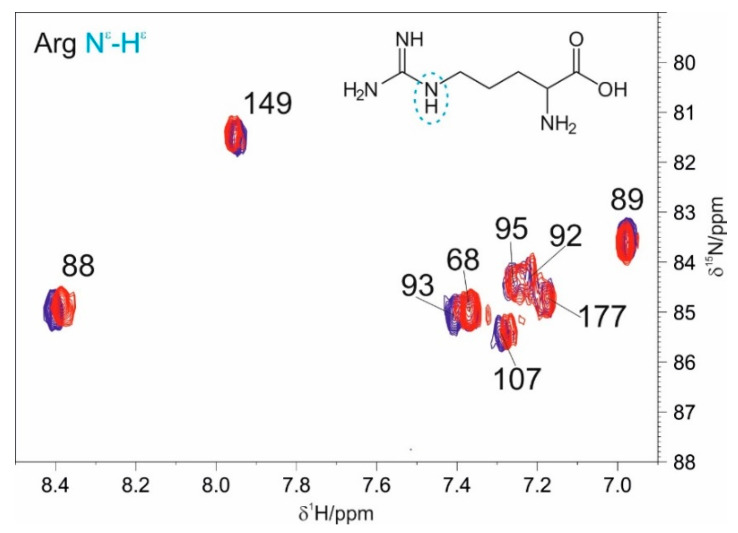
Overlay of 2D HN spectra recorded focusing on the arginine side chain region upon the addition of 1.2 equivalents of EP to NTD (blue reference, red addition of 1.2 equivalents of EP).

To assess the importance of positively charged residues in the interaction between NTD and EP, we also acquired a series of 2D HN-HSQC spectra centered in the region where the arginine side chain nitrogen nuclei are expected to resonate (δ^15^N ≈ 80 ppm). As reported in Figure 4, it is possible to observe that three out of nine H^ε^-N^ε^ signals were found to be perturbed (R88, R93, and R107) upon the addition of 1.2 equivalents of EP to the protein.

The protein fingerprinting can be expanded by performing a set of 2D ^13^C-detected NMR experiments (2D CON, 2D CACO, and 2D CBCACO) [54]. Preliminary to this, a 3D (H)CBCACON experiment [41] was also performed to complete the available assignment of NTD (BMRB: 34511 [22]). These experiments allowed us to assign 100% of the C^α^, C^β^, C′ 99.2% H^N^ (G44 missing), and 99.2% N (including those from the 11 proline residues, 8% of the total protein composition, G44 missing) resonances in our experimental conditions. Regarding side chains, we assigned also 100% of the H^ε^ and N^ε^ from arginine residues, and 24 out of 25 resonances arising from the side chains of glutamate, glutamine, aspartate, and asparagine residues [40] (Supporting Table S1, BMRB 51620). 

The analysis of the 2D CON spectrum shows a high heterogeneity in the intensities of the cross peaks (Figure S1). The most intense ones are those of the residues composing the initial and final protein’s regions (45-50 and 175-180) as well as part of the finger (92-106). This provides a qualitative but firm indication of the high flexibility of the basic finger, almost comparable to the initial and final residues within this domain. Analysis of the chemical shift perturbations (CSP) induced by EP confirms the picture achieved through 2D HN, highlighting a few additional peaks (L45, T49, H59, I94, D103, L104, N154, P162, L167, L169, and A173). Most importantly, the flexibility of the mobile tracts is maintained in the complex as one can verify by the intensities of the cross peaks in these regions also when 1.2 EP equivalents are added (Figure S1). The CACO/CBCACO experiments [40] provide information also on the C^β^ and C^α^ nuclei and on side chains containing carbonyl/carboxylate functional groups [40]. Major perturbations were identified for the residues, H59, I94, K100, L104, P162, and E174 (C^β^ and C^α^ resonances). Interestingly, H^δ^-C^δ^ and H^ε^-C^ε^ of H59 were found to be perturbed also in the 2D ^1^H-^13^C HSQC spectra acquired to monitor changes for the aromatic regions (data not shown). The region of carboxylate resonances of aspartate and glutamate residues in CACO spectra also shows interesting variations for the residues, E62, D63, D98, D103, and E174 (data not shown). 

**Figure 5:**
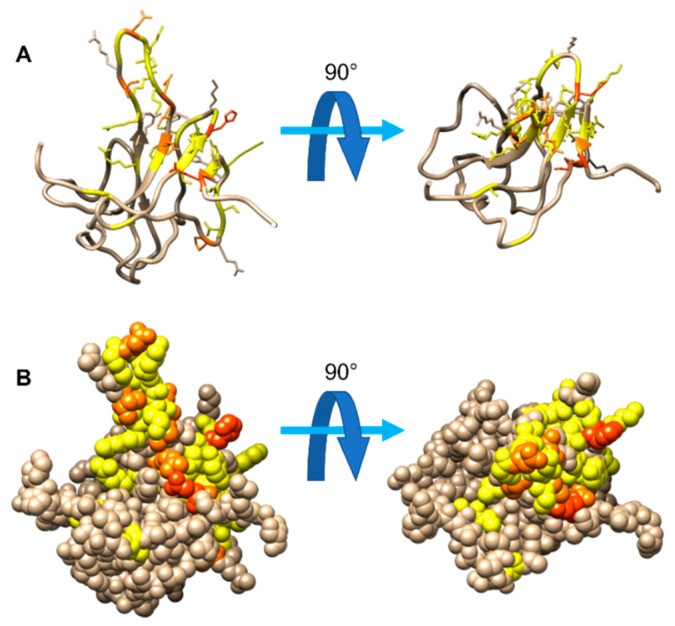
Mapping of the residues displaying the strongest perturbation in different 2D spectra (2D HN (backbone region), 2D HN (arginine region), 2D CON, 2D CACO, 2D CBCACO, and 2D HC (aromatic region)) at a molar ratio of 1:1.2 of NTD:EP. Panel (**A**) reports the protein in two different orientations, rotated by 90°, one with respect to the other; the heavy atoms of the perturbed residues are displayed as well. Panel (**B**) shows the same protein orientations with the models represented in a space-filling way. The color-coding is the following: residues which are found to be perturbed in a single experiment (yellow), in two experiments (orange), and in three or more experiments (red).

The collective analysis of these 2D NMR spectra provides a comprehensive view of the interaction of NTD with EP, reporting information on all backbone resonances and on selected side chain ones [40,54,55,56]. An overview of the most perturbed residues, considering all the analyzed spectra, is reported in Figure 5.

A more quantitative picture of the dynamic properties of NTD in the complex can be obtained through the analysis of the ^15^N relaxation rates (^15^N *R*_1_, ^15^N *R*_2_, and ^1^H-^15^N NOEs) for the isolated protein and upon the addition of 1.2 equivalents of EP (Figure S2). 

**Figure 6:**
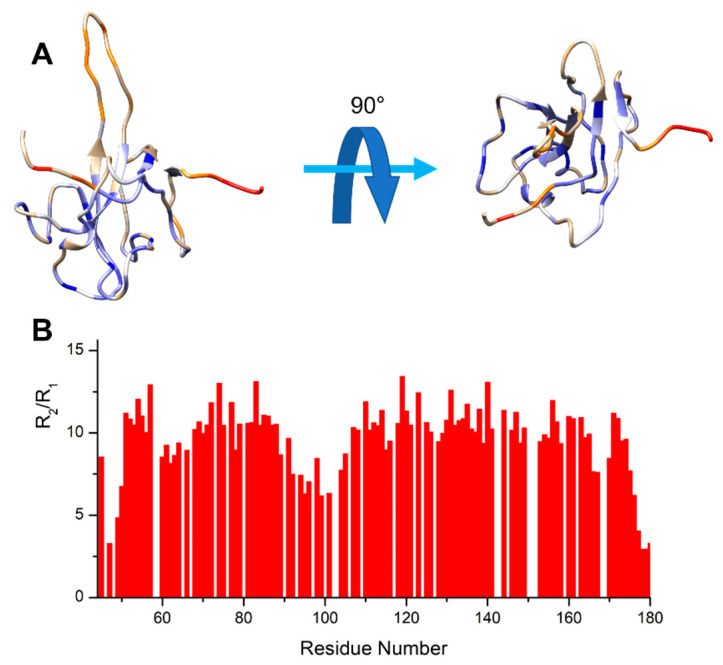
The NTD protein construct from two different views is reported in panel (**A**). The protein construct is colored on the basis of the *R*_2_/*R*_1_ values, which gives a first estimation of the correlation time. The residues with lower *R*_2_/*R*_1_ values are reported in red, while those with higher values are reported in blue. Panel (**B**) shows the *R*_2_/*R*_1_ values against the residue number.

The *R*_2_/*R*_1_ ratio (Figure 6) provides an initial estimation of the global correlation time. These values are mapped on the protein 3D model and reveal a more rigid core of the protein fold (blue, high *R*_2_/*R*_1_ values). On the other hand, several regions show higher flexibility (red, low *R*_2_/*R*_1_ ratios). These comprise the finger (residues 92-106), a few external loops, and the residues at the edges of the construct, as also previously reported in the literature [24,57,58].

The *R*_2_/*R*_1_ ratios can be used to estimate the local correlation time (τ_r_), as described in [47]. Focusing on the residues in the globular protein fold core (the blue ones in Figure 6), these are characterized by an average *R*_2_/*R*_1_ ratio value of 11.2 that provides a correlation time of 9 ns.

Upon interaction with EP, a homogeneous increase in the ^15^N *R*_2_ and ^1^H-^15^N NOE values is observed along with a reduction in the ^15^N *R*_1_ values (Figure S2). In this case, the *R*_2_/*R*_1_ ratio of the most rigid portion of the protein is 16.9. These variations are consistent with slower tumbling due to an increased molecular mass, which corresponds to a correlation time of 11.5 ns. Notably, even upon interaction, the flexibility of the finger, the loops, and the edges is maintained with lower *R*_2_/*R*_1_ ratio values with respect to the rest of the protein construct (Figure S2 Panel D).

Further evidence about the interaction can be achieved through DOSY experiments performed on the free and bound form of NTD, in the presence of 1.2 equivalents of EP (Figure S3). The obtained diffusion coefficients are D^FREE^: 1.5 ± 0.2 ·10^−10^ m^2^/s and D^BOUND^: 1.3 ± 0.1·10^−10^ m^2^/s. The smaller diffusion coefficient upon the addition of EP is in line with a reduced diffusion of the active species in solution.

Collectively, these observations support the presence of a quite extended surface of the interaction of NTD with EP and that the flexibility of the finger is retained in the complex.

To visualize the possible scenarios, we performed a docking calculation between the NTD construct and the EP molecule using the HADDOCK server [52,53]. The active residues were identified from all the previously mentioned observed CSP values (see the Materials and Methods section for details). 

**Figure 7:**
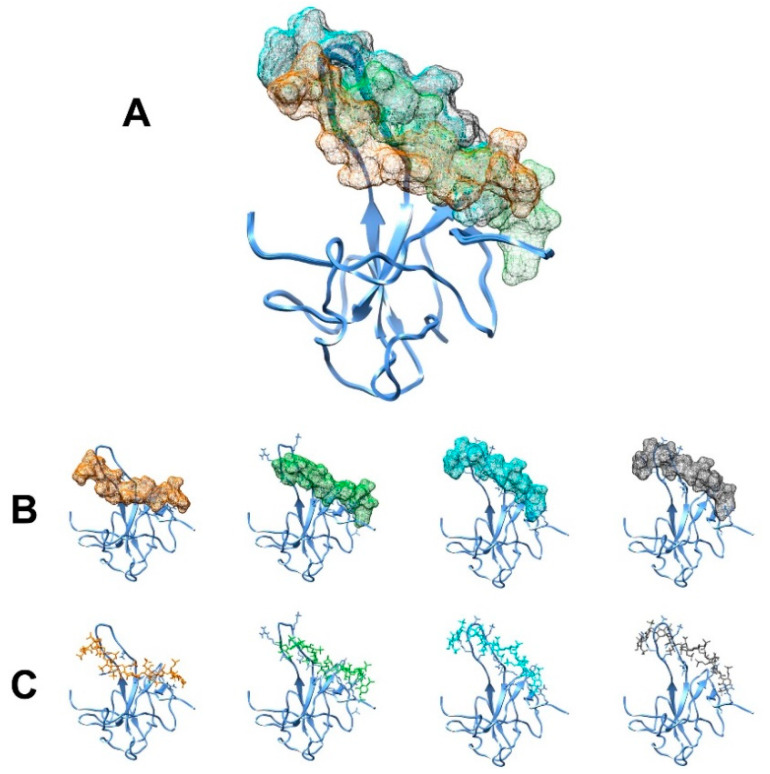
The results for the docking performed using HADDOCK are reported in the picture. The four structures derived from the best cluster are reported in Panels (**A**,**B**) in a superimposed and separate view, respectively. The protein structures are represented in the ribbon view while the mesh surface of EP is shown. The same four complexes with EP structure presented in stick view are displayed in Panel (**C**). Moreover, the side chains of NTD’s residues computed to be in close contact with EP are also shown in Panels (**B**,**C**).

Among the final 200 lowest-energy structures, 191 of them were divided into six clusters, with the one having the best HADDOCK score being selected for the analysis. This cluster, composed of 130 structures (68% of the total), possesses the best HADDOCK score (−49.7 ± 3.1) and provides the least violations of experimental restraints (144.2 ± 37.5 Kcal·mol^−1^). The four best representative structures of this cluster are reported in Figure 7.

As can be seen from panel A of Figure 7, the EP seems to surround the protein from the side of the β5 and β1 sheets, being in contact also with the region of the flexible finger. Looking in detail at the four best structural models (Figure 7, panels B and C), the residues computed to contribute most to the interaction with EP are I94, R95, G96, G97, K102, L104, and Y172. 

All the other clusters are found to have a lower HADDOCK score and higher violations of experimental restraints. In these clusters (Figure S4), the positive finger is always involved in the interaction. However, the core of the protein is computed to interact quite differently from cluster to cluster.

### 3.2. The Interaction of EP with NTR: The Role of the Intrinsically Disordered Regions 

Previous studies demonstrated the importance of the IDRs in enhancing the interaction potential of the N protein with its partners, such as RNA [16,17,19,23,24,59,60,61,62]. RNA can be considered as a polymer composed both of a negatively charged component (phosphodiester backbone groups) and an aromatic component (base groups). EP is also a linear polyanion with a strong negative charge and, in principle, it might mimic the charge properties of the RNA backbone. 

**Figure 8:**
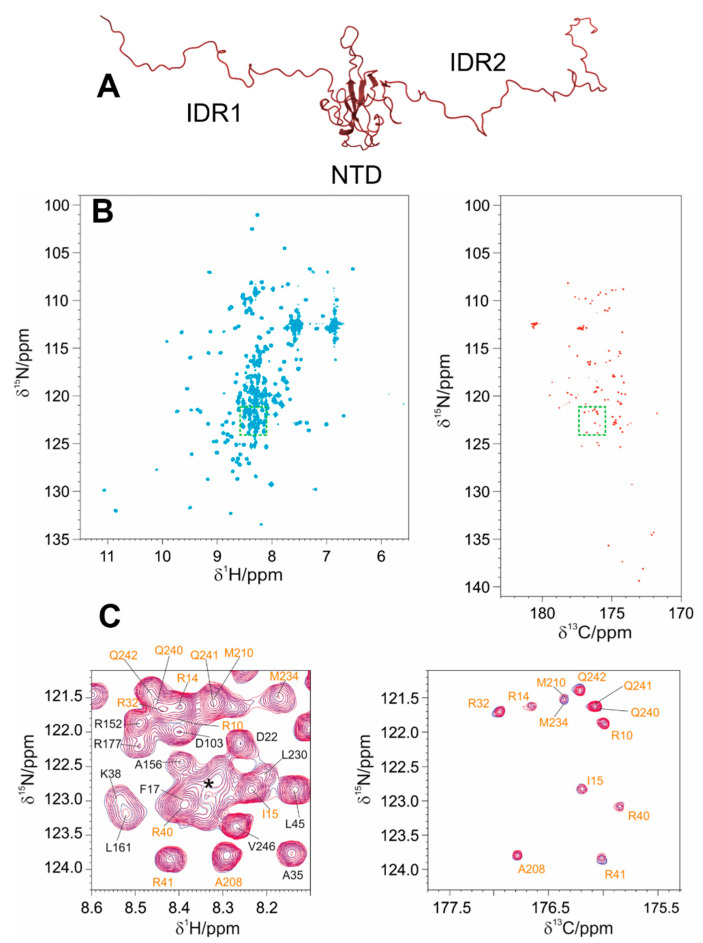
The picture reports the results of the mr_CON/HN [42] experiment Panels (**B**,**C**) performed on the NTR construct Panel (**A**). The 2D HN spectrum is reported on the left in Panel **B**; the 2D-CON spectrum is reported on the right in the same panel. Panel (**C**) shows a zoom of the superimposed spectra (HN in the left and CON on the right) acquired through the multiple receiver approach in the absence (blue) and with the addition of 0.1 equivalents of EP (magenta). These two regions are highlighted by green boxes in Panel (**B**). The CON provides superior resolution with respect to the HN one, which provides a higher number of signals, complicating the analysis.

We thus decided to assess how the two disordered regions flanking the globular NTD domain influence the interaction with EP by exploiting the NTR construct (1-248, IDR1-NTD-IDR2). To this end we opted for the mr_CON//HN [42] multiple-receiver NMR experiment, which allowed us to acquire two simultaneous NMR spectra of the protein, providing highly resolved information both for the globular domain and for the IDRs when part of the NTR construct (Figure 8). This experimental set-up is conceived to exploit the longitudinal recovery time necessary to restore the equilibrium of ^13^C magnetization for the 2D CON experiment to acquire the 2D HN FIDs needed for the 2D HN experiment. This experimental approach thus allows us to acquire the two spectra simultaneously, a key aspect to access experimental information on both globular domains and IDRs when part of a multidomain protein construct. 

From a more technical point of view, this strategy combines the sensitivity of the 2D HN experiment to pick up the signals arising from the globular domain with the high resolution provided by the 2D CON for the study of the IDRs. Indeed, this latter experiment acts as a relaxation filter that allows us to monitor the signals of the highly flexible regions in a clean way, enabling the study of IDRs within this modular construct rather than in isolation. 

**Figure 9:**
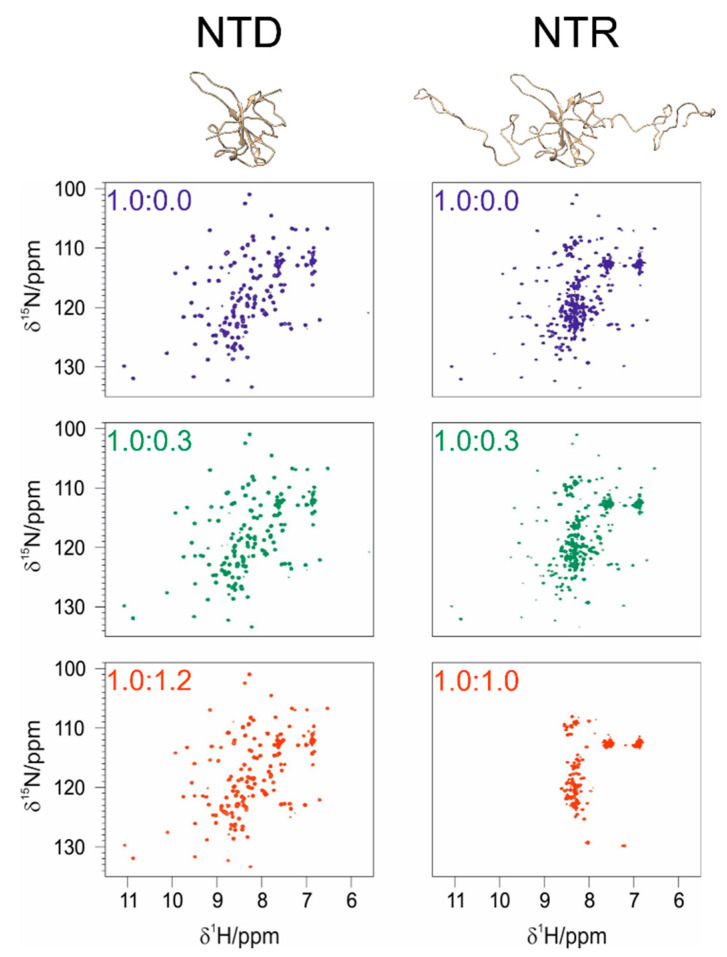
Comparison between the NTD and the NTR constructs at different molar ratios of protein:EP as reported in the top left corner of each spectrum.

A comparison of the 2D HN spectra of NTD and NTR with a comparable protein:EP molar ratio is reported in Figure 9 and shows that the IDRs have a marked effect on EP binding. Focusing on the well-dispersed signals of the globular domain in the NTR construct, these show similar chemical shift perturbations as those observed when studying the isolated NTD construct (Figure S5). However, a pronounced decrease in the intensities of the cross peaks of the globular domain is also observed even in the presence of low amounts of EP (1:0.3 NTR:EP, Figure 9 and Figure S5). This leads to the complete disappearance of the cross-peaks from the globular domain at the molar ratio of 1:1, while a set of cross peaks deriving from the IDRs is still observed. The extensive broadening of the cross peaks of the globular domain is probably due to the increased molecular mass and structural heterogeneity of the NTR construct with respect to the NTD one, which implies a slower tumbling upon interaction, with the IDRs still retaining their flexibility [56]. Indeed, the addition of 110 flexible amino acids further increases the structural complexity of the protein. IDR1 and IDR2 highly extend the conformational space sampled by the protein. The occurrence of intermolecular interactions mediated by EP promoting the increase in the molecular mass cannot be ruled out [57].

It is interesting to inspect the perturbations sensed by the IDRs upon interaction with EP at the residue level. Most of the resonances arising from the IDRs fall in crowded regions in the 2D HN spectra, complicating the analysis (Figure 8 panel C). However, some cross peaks show measurable CSP values, as shown in Figure S6, including residues in the proximity of the NTD (e.g., S183 in Figure S6). The fitting of the CSP values measured for the few resolved peaks observed all along the titration provides *K_d_* = 8 ± 3 µM (L45 and S176). A value in the same range, *K_d_* = 10.6 ± 0.4 µM, was obtained by Isothermal Titration Calorimetry (ITC) (Figure S7) which was used to corroborate the NMR-derived result. The IDRs appear thus responsible for a higher affinity of the NTR construct for heparin.

**Figure 10:**
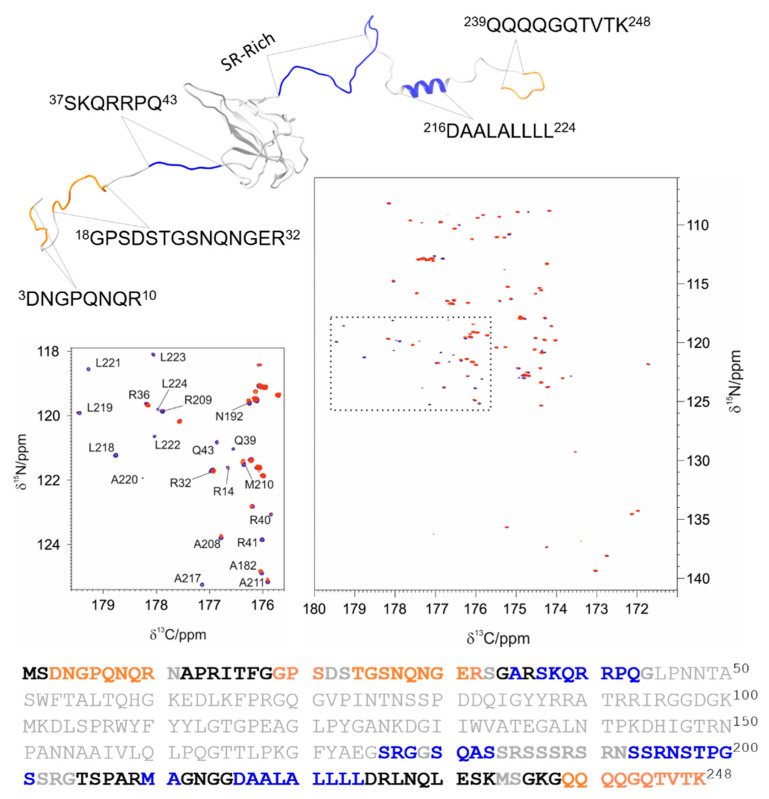
The picture reports the superimposition of the two CON spectra of NTR: the spectrum before the addition of EP (blue) and the one acquired after the addition of 0.3 equivalents of EP (red). The positively charged regions of the protein and the poly-Leu region are found to be the most affected by the interaction. This is highlighted in the expansion reported on the left, where the cross peaks from nuclei in the stretches 36-43, 208-211, and 216-224 are shown. A structural model of NTR is reported in the upper part of the figure and the primary sequence of the protein in the lower part. The IDRs are reported in bold, and the following color coding has been used to highlight the different behavior of specific tracts: the residues that are still observable at the end of the titration are reported in orange, while the residues that show a variation in chemical shift and/or a reduction in intensity are reported in blue.

Inspection of the CON allows us to clearly focus on the signals of the residues in the IDRs. Figure 10 shows an overlay of the CON spectra before and after the addition of 0.3 equivalents of EP. The spectra clearly show that a subset of cross peaks experiences a reduction in intensity (a few cross peaks experience also minor chemical shift changes). A plot of the intensity ratios versus the residue number is shown in Figure S8. There are two main regions showing a significant decrease in signal intensities. The first is the portion ^36^RSKQRRPQ^43^, whose signals completely disappear. A second interesting region is the so-called poly-Leu region, characterized by the residues ^216^DAALALLLL^224^. All the peaks that belong to this region disappear upon the addition of 0.3 EP equivalents. Several residues in the initial part of IDR2 are also perturbed. It is also interesting to note which residues are still observable upon the final addition of EP. These are mainly in the final region of IDR2 (^239^QQQQGQTVTK^248^) and two regions of IDR1 (^3^DNGPQNQR^10^ and ^24^TGSNQNGE^31^).

Interestingly a subset of cross peaks shows a higher intensity in the presence of EP (Figure S7). These are due to the nuclei of residues in regions that remain highly flexible in the complex and are almost all “disorder-promoting” amino acids [63,64,65,66,67], with a large share of glycine and glutamine residues. The increase in intensity upon binding could be related to the increased mobility of these residues in the complex with respect to that in the isolated protein [68]. 

## 4. Discussion

### 4.1. The Dynamical Binding Modes of NTD 

The combined analysis of the CSP determined through the 2D NMR spectra based on ^1^H^N^- and ^13^C’-detection delineates a clustering in two main regions on the NTD, the basic finger and the β1–β5 antiparallel sheet. The basic finger is mainly characterized by positively charged residues (5 out of 15 residues in the 92-106 stretch) and possesses an amino acid pattern characteristic of a glicosaminoglycan (GAG)-binding domain (an X-BXBX motif in the ^104^LDKMKG^99^ region) [69,70]. This region is found to be perturbed in our analysis (99-101-102 perturbed in the 2D HN spectra, 101-102-103-104 perturbed in the 2D CON spectra, and 100-104 perturbed in the 2D CBCACO). Interestingly, this region contains two lysine residues (K100, K102) but does not possess any arginine residue, usually the primary actors in a protein–GAG interaction. However, the side chains of arginine residues very close to this main interaction site (99-104) were found to be perturbed. Indeed, the resonances of H^ε^-N^ε^ of R93 and R107 are affected upon the addition of EP to the protein solution. The involvement of the finger in the interaction with EP is thus in line with predictions/expectations. On the contrary, it is interesting to note that most of the residues forming the β1 and β5 secondary structure elements are not positively charged (β1: ^56^LTQ^58^ and β5: ^171^FYA^173^), nor is the region preceding β5 (^165^TTLPK^169^), which is also perturbed upon the addition of EP. In addition, E62 and D63, in the loop following strand β1 and E174 at the end of strand β5 are also perturbed. These are negatively charged and are likely to be engaged in intramolecular electrostatic interactions. Therefore, the observed changes in these regions upon the addition of EP could also be due to perturbations that are propagated throughout the 3D structure. Indeed, the β1 and β5 strands are very short, and β5 is close to the terminal amino acid of the NTD domain, two aspects that render this region quite sensitive to perturbations, a change that could then be easily propagated to the preceding residues (165-169). 

The highly negatively charged compound EP could be driven to the positively charged basic finger of NTD thanks to the strong electrostatic attraction. However, its dimension (16mer, 4.5 KDa) and the absence of hydrophobicity limit the contact with the protein core. On the other hand, the bulkiness of EP could play an important role in perturbing the structure close to the domains’ ends, also interfering with the network of intramolecular electrostatic interactions. Thus, even residues located far from the initial interaction surface can be perturbed due to structural fluctuations.

The docking analysis supports this picture. Considering the best results of the docking, the interaction region is overall positively charged comprising the highly charged region of the finger. HADDOCK computes electrostatic force as the main contribution of the interaction (−380.6 ± 83.4 Kcal·mol^−1^) with respect to the Van der Waals energy (−38.6 ± 5.7 Kcal·mol^−1^). This is in line with the opposite charges of the two interacting partners. Additionally, from the docking point of view, the interaction between EP and the protein core is hindered, with EP placed on the edge of the NTD’s surface capable of disrupting intramolecular interactions that eventually occur, as well as possible intermolecular interactions with other partner molecules such as RNA.

The binding affinity between the two molecules and the peculiar folding topology of the protein limit the representation of the binding with a unique, well-defined binding model and indicate an extended perturbed surface. In this representation, the basic residues are the main drivers of the interaction and imply structural modifications sensed far from the binding region.

This is also supported by the analysis of the dynamic properties of NTD. In the presence of EP, the relaxation properties are indicative of a species with higher molecular mass in solution, with increased *R*_2_/*R*_1_ ratios; flexibility in the finger is retained upon binding. This is a typical behavior of modular proteins in a transient complex with RNA [71].

It is interesting to compare our results with recent studies focusing on the interaction of NTD with different fragments of nucleic acids. Indeed, NMR spectra were used to follow CSPs of NTD upon the addition of increasing concentrations of nucleic acid fragments to map interaction surfaces [19,22,25,58,72]. The region of the basic finger is generally extensively perturbed. Interestingly, the mutation of R92 was found to abrogate the interaction with DNA [73]. Another common feature monitored in these studies is that the perturbed residues are not limited to a specific region of the protein, but generally, large surface areas are found to be perturbed upon interaction. The interaction with EP significantly resembles this general behavior, indicating that it shares common features with the interplay of NTD with different kinds of polyanions, such as nucleic acid fragments. The identification of specific features linked to the different types of partner molecules (RNA, DNA), to whether they are single or double strand, to how the length of the fragment and its conformation affect the interaction are still a matter of debate [22,25,58].

### 4.2. The Role of IDRs in Orchestrating NTR-EP Interaction

The important role of the flexible linkers in modulating the properties of N has been pointed out in the literature since early studies on the SARS-CoV-1 variant that showed how the linkers promote an increase in the affinity of the NTD for fragments of RNA [16]. However, atom-resolved information about their role has remained elusive, as only recently a sequence-specific assignment of the linkers in the context of the NTR construct has become available [15,21]. Briefly, ^13^C-direct detection has been recently demonstrated to be an effective tool to monitor the effect of IDRs in the interplay between gRNA and NTR [19]. This now allows us to inspect in detail the effect of the IDRs also on the interaction with EP. The IDRs comprise the majority of the basic amino acids distributed along the primary sequence of the protein (13 arginine and four lysine residues). This class of amino acids can be the first interacting partners to a negatively charged molecule such as EP [74]. Moreover, the high mobility typical of IDRs facilitates the encounter between the two molecules with a much higher sampled space with respect to the NTD finger [75,76]. This is in line with the increased affinity for EP observed when the two IDRs flank the NTD in the NTR construct.

Zooming into the IDRs through 2D NMR spectra, in particular through the 2D CON experiments, which reveal atom-resolved information about the IDRs in a very clean way [19], it is interesting to note that different regions of the IDRs are perturbed to different extents. In particular, two arginine-rich regions are significantly perturbed, in agreement with the electrostatic sensing of negatively charged EP. Interestingly the most perturbed segment in IDR1 (^37^SKQRRPQ^43^) has a characteristic EP interaction motif [69]. However, the overall picture is more complex than that, as expected from a structurally and dynamically heterogenous protein such as the NTR [76,77,78]. For example, the addition of EP also influences the resonances in the 216–225 region, a quite remote one from the NTD. This region, mainly composed of leucine residues (^217^AALALLLL^224^), adopts a helical conformation and is flanked by two aspartate residues (D216 and D225), all features that render it quite inappropriate for direct interaction with the highly negatively charged “ligand”. The observed changes could thus be due to the disruption of intra-molecular interactions that are perturbed by the interaction with EP, as also observed upon interaction with RNA [19]. Finally, other observed features upon binding are that a subset of the residues of the IDRs remain highly flexible; actually, in a few cases, they even seem to increase their mobility upon interaction. This could result from the perturbation of the ensemble of the conformers describing the NTR when in the presence of EP. The amino acids whose mobility is enhanced upon the addition of EP (residues Q4, P6, Q7 N8, Q9, F17, P20, T24, G44, G200, L201, G238, and Q239) mainly belong to the so-called “disordered promoting” class (A, R, G, Q, S, P, and E), in particular, with several glycine and glutamine residues involved in the peptide bond (6 and 5, respectively) [79,80,81]. Interestingly, compensatory adaptations in different regions of an IDP, osteopontin, were previously observed upon interaction with heparin [82], reminiscent of the observations in the present study. 

These new insights provide a hint of the stronger anchoring of EP on the extended protein surface of NTR. The IDRs, together with the basic finger, seem to act as *sensors* for negatively charged molecules. They might create a platform to accommodate the long polysaccharide on NTD while exploiting a major surface area given by the disordered regions. Moreover, the structural and dynamic features induced by IDRs further complicate the binding mode landscape, as often happens when IDPs/IDRs are involved in binding [75,81,83,84,85].

In particular, the exposed arginine residues scattered on the primary sequence of flexible regions not only establish strong coulombic interactions but they can also participate in hydrogen bonds with the sulfate groups of the EP. The distribution of the charged residues, in particular in the SR-motif, could simulate the effect of GAG-binding motifs, determining the stronger affinity observed for NTR. Serine residues are indeed the most frequent amino acids which intercalate the cluster of basic residues typically observed in many heparin-binding motifs [69].

## 5. Conclusions

We have shown that the N-terminal region of N from SARS-CoV-2 (NTR, 1-248) interacts with enoxaparin. This interaction was initially investigated by focusing on the NTD globular domain (44-180). This allowed us to map on the 3D structure of this domain an extended region perturbed upon the addition of EP, with the core of the interaction being the flexible basic finger, rich in positively charged residues. As a following step, we showed that two disordered regions flanking the globular domain, IDR1 (1-45) and IDR2 (181-248), contribute to an increase in the affinity of EP to the protein. NMR allowed us to access atom-resolved information on the two IDRs part of the whole NTR construct, revealing a complex interplay between different regions of this multi-domain protein construct and highlighting the importance of these flexible segments for the protein behavior when an interaction occurs. Selected motifs on the IDRs, rich in arginine residues, were shown to be involved in the interaction. Interestingly the data also reveal protein regions that remain highly flexible in the complex.

These molecular details on the interaction of N with EP may contribute to understanding the possible interactions of N with endogenous heparin/glycosaminoglycans, as well as to reveal unpredicted roles exerted by low molecular weight heparin used in the treatment of COVID-19. The perspective of this work is the investigation of the full-length N protein, which can provide further insights into understanding the key mechanism of the interaction of the protein with polyanions able to interfere with its function.

## Figures and Tables

**Table 1:** Acquisition parameters for the recorded spectra.

Construct	Experiment	Data Points		Spectral Width (Hz)		Number of Scans	Interscan Delay (s)	Field (^1^H MHz)
		F1	F2	F1	F2			
NTD	2D CACO	128	1024	7407 (^13^C^α^)	5263 (^13^C′)	32	1.6	700
NTD	2D (H)CBCACO	174	1024	11,628 (^13^C^ali^)	5263 (^13^C′)	32	1.0	700
NTD	2D (HCA)CON	128	1024	3413 (^15^N)	5000 (^13^C′)	96	1.1	700
NTD	2D HC	256	1024	10,638 (^13^C^aro^)	11,364 (^1^H)	4	1.1	700
NTD	2D HN	256	2048	4347 (^15^N)	19,132 (^1^H)	16	1.0	950
NTD	2D H^ε^N^ε^	256	4096	11,627 (^15^N^ε^)	19,132 (^1^H^ε^)	8	1.0	950
NTR	mr_**CON**//HN	400	1024	2840 (^15^N)	5263 (^13^C)	16	1.9	700
NTR	mr_CON//**HN**	400	4096	3195 (^15^N)	20,833 (^1^H)	32	1.9	700

For the mr_CON//HN, the experiment to which the parameters are referred is in bold.

## Data Availability

The data presented in this study are available upon request from the corresponding authors. The obtained NMR assignment has been deposited on the BMRB under the accession code 51620.

## Supplementary Materials

The following supporting information can be downloaded at: https://www.mdpi.com/article/10.3390/biom12091302/s1, Table S1: assigned resonances; Figure S1: CON peaks intensity for the NTD construct, Figure S2: ^15^N R_2_, R_1_, NOE values, Figure S3: DOSY spectra, Figure S4: Cluster 2 to 6 obtained from HADDOCK, Figure S5: CSP and Intensity ratio for NTD and NTR, Figure S6: ITC titration, Figure S7: zoom of HN spectra for the NTR construct, Figure S8: Intensity ratios of CON’s peaks.
