# Supplementary material for: The Role of Disordered Regions in Orchestrating the Properties of Multidomain Proteins: The SARS-CoV-2 Nucleocapsid Protein and Its Interaction with Enoxaparin
Source: Biomolecules. 2022 Sep 15;12(9):1302. doi: 10.3390/biom12091302 (PMC9496478; doi:10.3390/biom12091302)

The Role of Disordered Regions in Orchestrating the Properties of Multidomain Proteins: The SARS-CoV-2 Nucleocapsid Protein and Its Interaction with Enoxaparin

Marco Schiavina †, Letizia Pontoriero †, Giuseppe Tagliaferro, Roberta Pierattelli * and Isabella C. Felli *

Magnetic Resonance Center (CERM) and Department of Chemistry "Ugo Schiff", University of Florence, Via L. Sacconi 6, 50019 Sesto Fiorentino (Florence), Italy

† These authors contributed equally to the work.
* Correspondence: roberta.pierattelli@unifi.it (R.M.); felli@cerm.unifi.it (I.C.F.)

Supplementary Tables

Table S1: Chemical shift values (ppm) for the newly assigned ^{13}C and ^{15}N resonances of the NTD construct in 25 mM KH_2PO_4/K_2HPO_4 buffer, 150 mM KCl, pH 6.5 at 298K. This chemical shift values has been deposited on the BMRB code under the accession code 51620.

Assignment	δ (ppm)
N46-PRO	135.90
C^{γ}47-ASN	177.03
C^{γ}48-ASN	177.30
C^{γ}62-GLU	33.59
C^{δ}62-GLU	183.93
C^{γ}63-ASP	179.34
N67-PRO	135.50
C^{γ}70-GLN	33.44
C^{δ}70-GLN	178.77
N73-PRO	140.31
C^{γ}75-ASN	177.29
C^{γ}77-ASN	177.69
N80-PRO	140.43
C^{γ}81-ASP	178.52
C^{γ}82-ASP	179.18
C^{γ}83-GLN	32.44
C^{δ}83-GLN	180.38
C^{γ}98-ASP	180.15
C^{γ}103-ASP	180.18
N106-PRO	138.21
N117-PRO	134.29
C^{γ}118-GLU	33.989
C^{δ}118-GLU	181.22
N122-PRO	137.26
C^{γ}126-ASN	176.86
C^{γ}128-ASP	179.87
C^{γ}136-GLU	35.79
C^{δ}136-GLU	183.74
C^{γ}140-ASN	176.06
N142-PRO	132.72
C^{γ}144-ASP	179.13
C^{γ}150-ASN	177.39
N151-PRO	136.92
C^{γ}153-ASN	175.91
C^{γ}154-ASN	177.19
C^{γ}160-GLN	33.97
C^{δ}160-GLN	180.91
N162-PRO	133.48

C^{γ}163-GLN	33.47
C^{δ}163-GLN	180.25
N168-PRO	136.06
C^{γ}174-GLU	36.29
C^{δ}174-GLU	183.55

Supplementary Figures

Figure S1: The intensities of the cross peaks in CON spectra of NTD are reported versus the residue number for the isolated protein (blue) and after addition of 0.3 equivalents of EP (red).

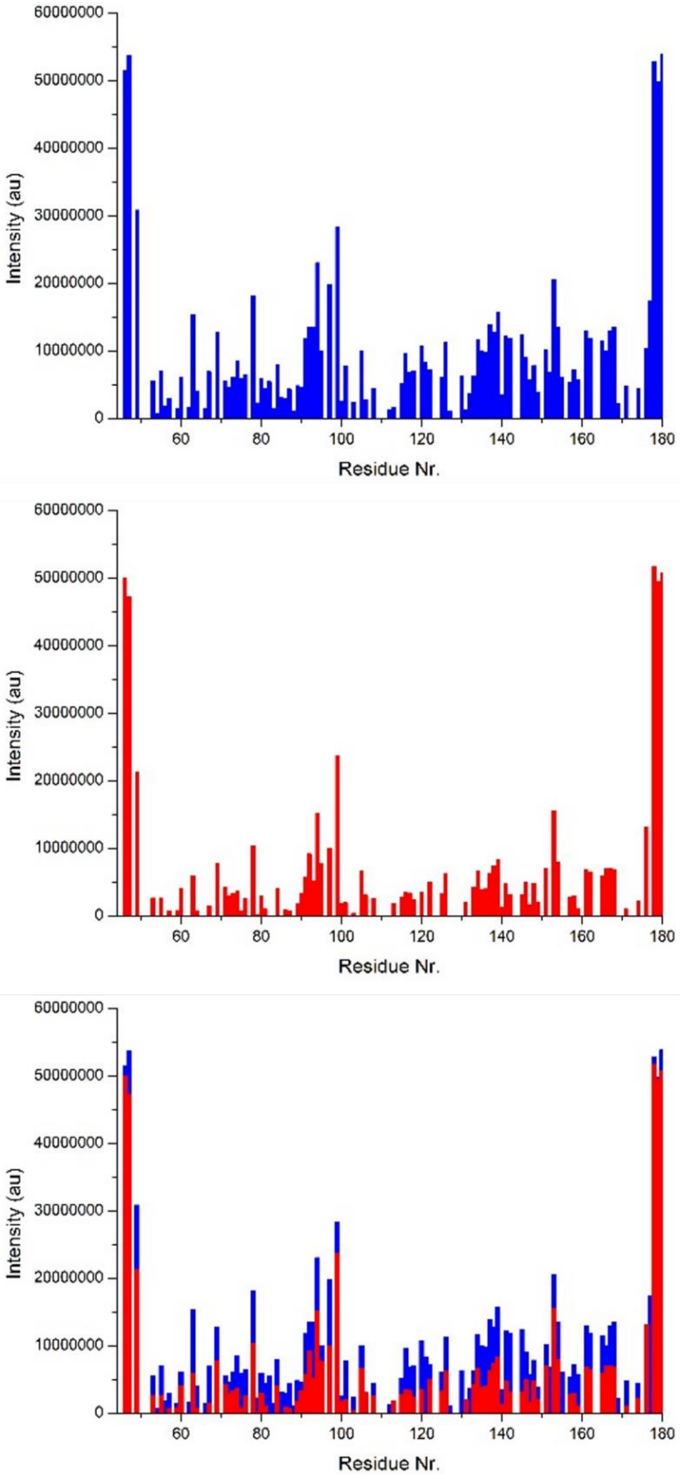

Figure S2: The central region involved in the interaction with EP is the most flexible one in the NTD construct. Upon EP binding, the mobility is overall reduced with the flexible regions that, however, still retain their flexibility. The protein possesses a flexible loop, the basic finger, which spans from residue 92 to 106.The ^{15}N R_2, R_1, NOE values are reported in panel A for the free form. Panel B reports the same values for the bound form. In panel C is reported the overlay of the ^{15}N R_2, R_1 and NOE values. R_2/R_1 values are reported for the bound form against the primary sequence in panel D.

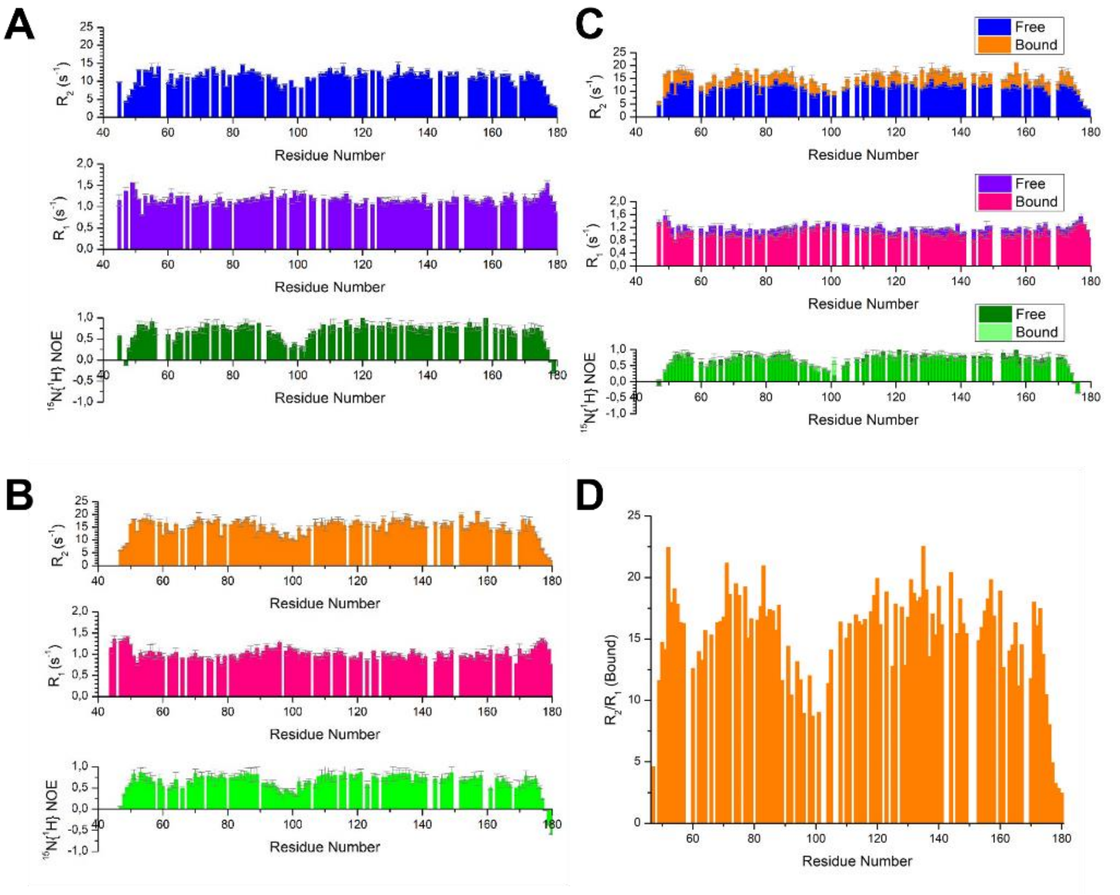

Figure S3: DOSY experiments [48] confirm the binding of heparin to NTD. The data obtained for the NTD protein alone are reported in blue, the ones determined for the NTD:EP adduct (1:9.6) are reported in magenta. The NTD:EP adduct has a slower diffusion time with respect to NTD as it can be appreciated comparing the dotted lines (light blue for the free form (upper), green for the bound form (lower)).

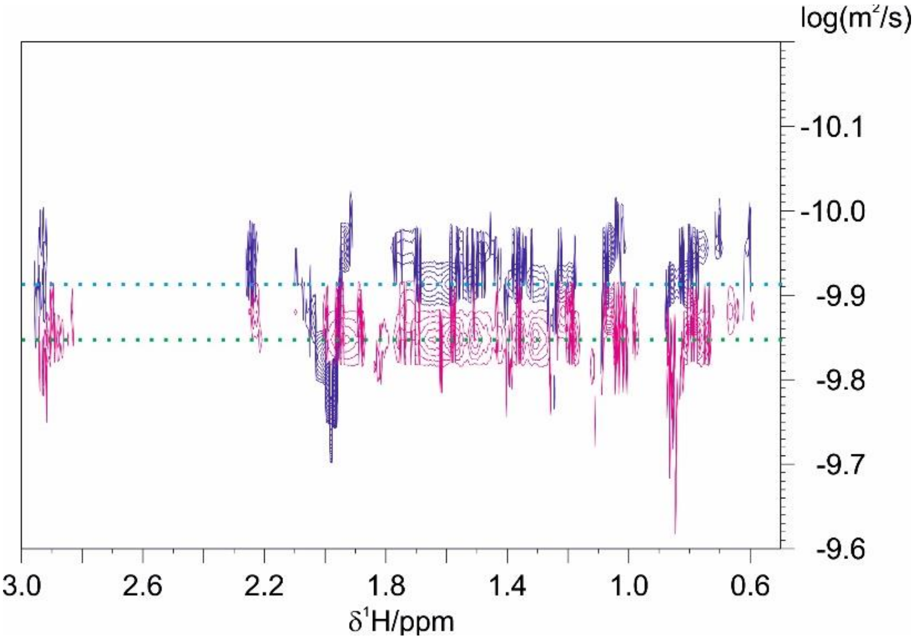

Figure S4: The four best structures of clusters 2 – 6 derived from the docking are reported in the picture. All these clusters present a HADDOCK [52,53] score which is lower with respect to Cluster 1.

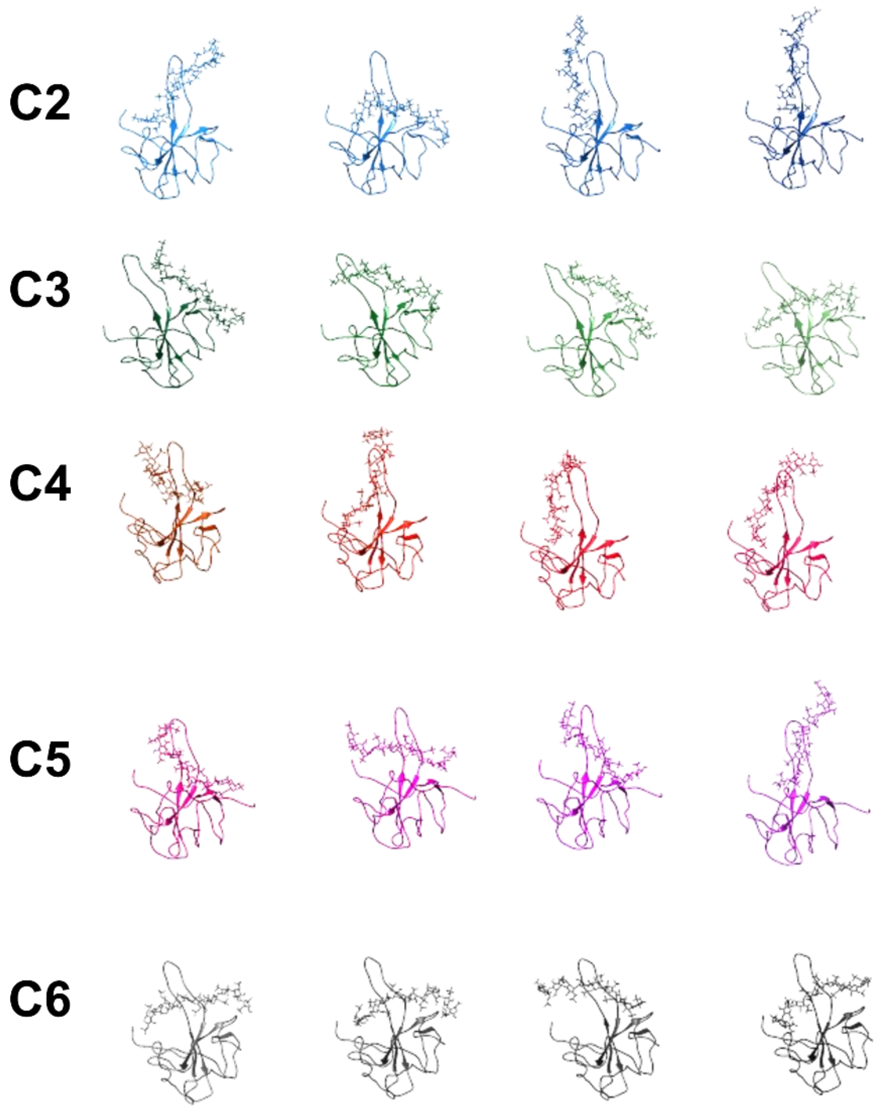

Figure S5: Comparison of CSP and intensity ratio for the two N constructs at the same protein:EP ratio (equal to 1:0.3). The data for NTD are reported on the left, the ones for NTR on the right.

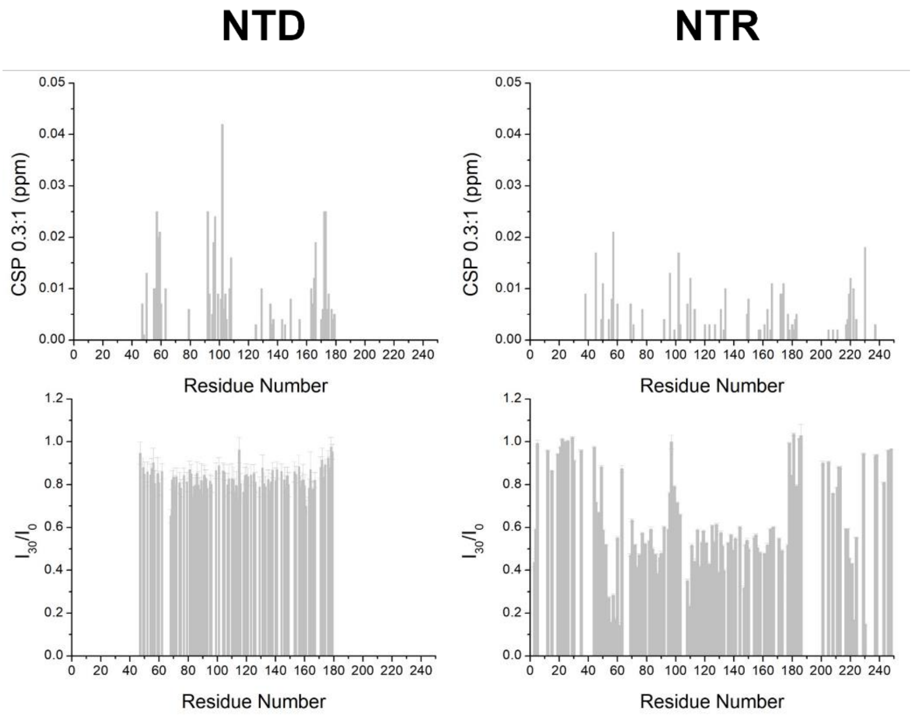

Figure S6: Panel A reports the overlay of NTR 2D HN spectra upon addition of EP. Blue, red, green, cyan and pink represent the 1:0, 1:0.1, 1:0.3, 1:06 and 1:1.2 molar ratio of NTR:EP respectively. The residues closest to the globular domain (NTD) experience the strongest perturbation in term of CSP and decrease in intensity (S176, S180, S183). Other resonances from residues belonging to the IDR2 are found to be perturbed as well (S201, T205). On the other hand, peaks in the initial part of IDR1 are not perturbed at all (S21, T24, S26). A subset of peaks falls in a very crowded region (ca. 116.5 ppm ^{15}N), where the resolution of 2D HN spectra is not enough to achieve information at the residue level.
Panel B reports the fitting of the K_d obtained from the CSP analysis for L45 and S176, presenting a K_d of 8 ± 3 μM

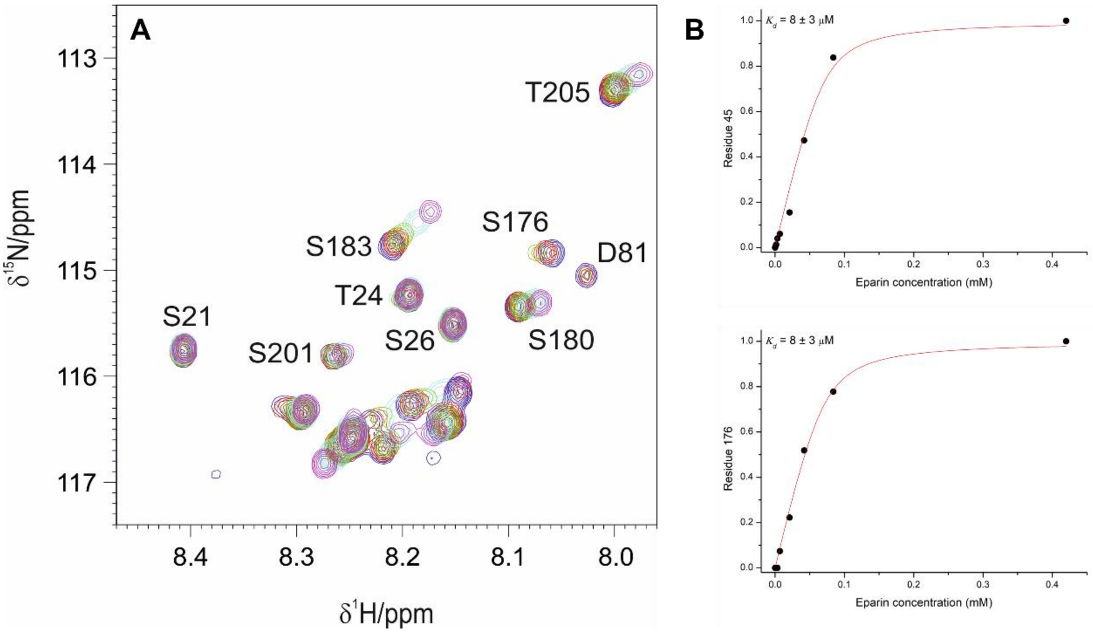

Figure S7: ITC experiments confirms a higher affinity for NTR with respect to NTD. The K_d of the interaction can be estimated in the order of 10 μM.

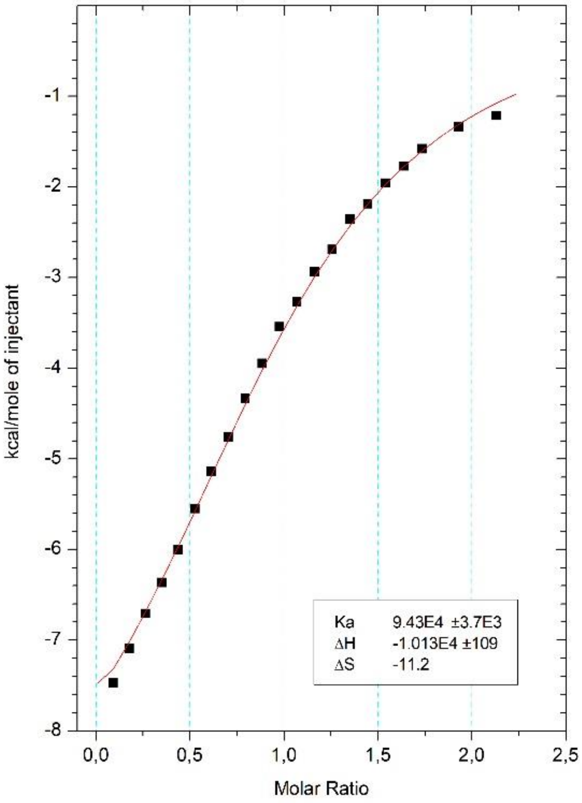

Figure S8: Intensity ratios of cross peaks observed in 2D CON NMR spectra acquired with increasing amounts of EP.
A subset of cross peaks shows a higher intensity in the presence of EP, in particular $^{3}DQ^{4}$, $^{5}GP^{6}$, $^{8}NQ^{9}$, $^{19}GP^{20}$, $^{43}QG^{44}$, $^{199}PG^{200}$, $^{204}GT^{205}$, $^{237}KG^{238}$ and $^{238}GQ^{239}$ considering that the CON experiment reveals C'_{i-1}-N_i correlations. These are almost all "disorder-promoting" amino acids, with a large share of glycine and glutamine residues [79-81].

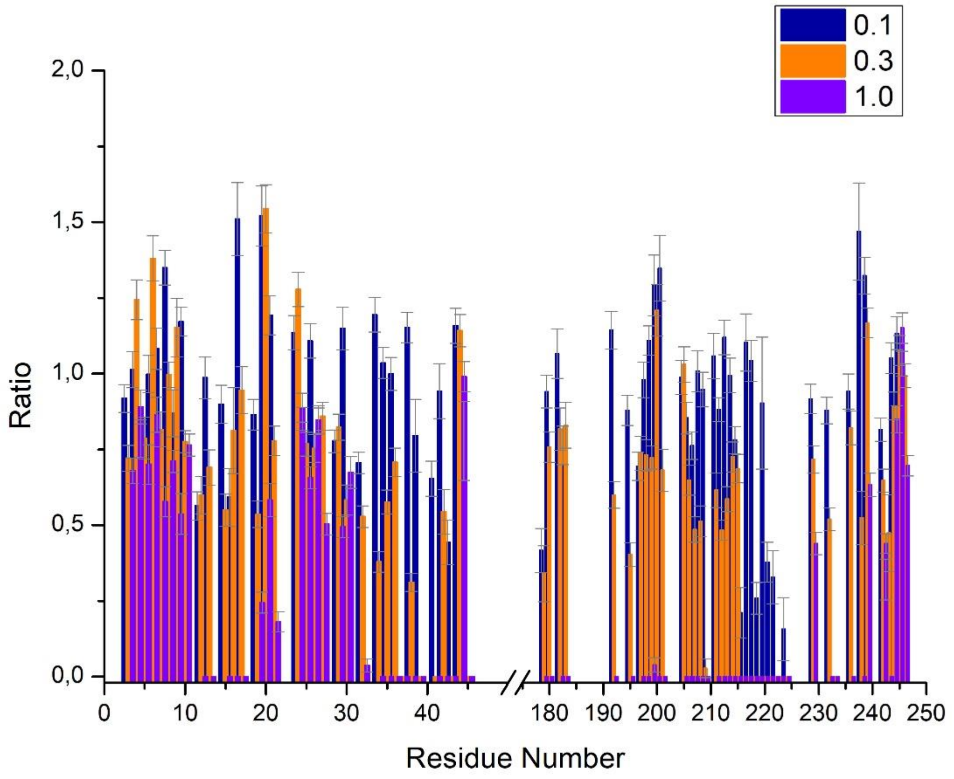

Supplement: Supplementary file 1 [file biomolecules-12-01302-s001.zip › biomolecules-1861058-supplementary.pdf]
